# Genome-Wide Association Mapping of Seedling Drought Tolerance in Winter Wheat

**DOI:** 10.3389/fpls.2020.573786

**Published:** 2020-10-28

**Authors:** Frank Maulana, Wangqi Huang, Joshua D. Anderson, Xue-Feng Ma

**Affiliations:** ^1^Noble Research Institute, LLC, Ardmore, OK, United States; ^2^State Key Laboratory for Conservation and Utilization of Bio-Resources in Yunnan, Yunnan Agricultural University, Kunming, China

**Keywords:** drought stress, GWAS, QTL, seedling stress, wheat

## Abstract

In the southern Great Plains of the United States, winter wheat grown for dual-purpose is often planted early, which puts it at risk for drought stress at the seedling stage in the autumn. To map quantitative trait loci (QTL) associated with seedling drought tolerance, a genome-wide association study (GWAS) was performed on a hard winter wheat association mapping panel. Two sets of plants were planted in the greenhouse initially under well-watered conditions. At the five-leaf stage, one set continued to receive the optimum amount of water, whereas watering was withdrawn from the other set (drought stress treatment) for 14 days to mimic drought stress. Large phenotypic variation was observed in leaf chlorophyll content, leaf chlorophyll fluorescence, shoot length, number of leaves per seedling, and seedling recovery. A mixed linear model analysis detected multiple significant QTL associated with seedling drought tolerance-related traits on chromosomes 1B, 2A, 2B, 2D, 3A, 3B, 3D, 4B, 5A, 5B, 6B, and 7B. Among those, 12 stable QTL responding to drought stress for various traits were identified. Shoot length and leaf chlorophyll fluorescence were good indicators in responding to drought stress because most of the drought responding QTL detected using means of these two traits were also detected in at least two experimental repeats. These stable QTL are more valuable for use in marker-assisted selection during wheat breeding. Moreover, different traits were mapped on several common chromosomes, such as 1B, 2B, 3B, and 6B, and two QTL clusters associated with three or more traits were located at 107–130 and 80–83 cM on chromosomes 2B and 6B, respectively. Furthermore, some QTL detected in this study co-localized with previously reported QTL for root and shoot traits at the seedling stage and canopy temperature at the grain-filling stage of wheat. In addition, several of the mapped chromosomes were also associated with drought tolerance during the flowering or grain-filling stage in wheat. Some significant single-nucleotide polymorphisms (SNPs) were aligned to candidate genes playing roles in plant abiotic stress responses. The SNP markers identified in this study will be further validated and used for marker-assisted breeding of seedling drought tolerance during dual-purpose wheat breeding.

## Introduction

Winter wheat (*Triticum aestivum* L.) is the most important cereal crop grown for forage and grain production in the southern Great Plains of the United States (Kumssa et al., 2019). In this region, when wheat is grown for winter pasture, it is often planted at least 2–3 weeks earlier than wheat grown for grain-only production. However, early planting of the crop increases the risk of establishment failure due to potential drought stress in the autumn (Maulana et al., 2019). Drought stress at the seedling stage has been shown to reduce the photosynthetic activity and respiration rate of seedlings, resulting in death of seedlings due to excessive dehydration (Dhanda et al., 2002; Sallam et al., 2019).

To date, significant attempts have been made to improve resilience against drought stress by conventional breeding in different crop species, including wheat. However, improving drought tolerance using conventional approaches has been proven difficult because of the genetic complexity of the trait, which is contributed by multiple quantitative trait loci (QTL) (Edae et al., 2014; Gupta et al., 2017; Wang and Qin, 2017; Lehnert et al., 2018). Besides, it is difficult to phenotype drought tolerance under field conditions, especially when plants are at the seedling stage.

Different morphological and physiological traits respond to drought stress. Therefore, breeding for drought tolerance requires characterization of plant morphological and physiological traits that are associated with drought tolerance, so that genes controlling those traits can be identified for marker-assisted selection (MAS). Leaf chlorophyll content and leaf chlorophyll fluorescence have been shown to be associated with drought stress response in plants, and these traits can be used to select drought-tolerant genotypes under drought stress conditions (Jiang et al., 2004; Li et al., 2006). Studies have shown positive correlations between physiological traits, such as leaf chlorophyll content and leaf chlorophyll fluorescence, and yield traits, such as grain yield (GY) and yield components, under well-watered (WW) and drought-stressed (DS) growth conditions in wheat (Pinto et al., 2010) and sorghum (*Sorghum bicolor* L. Moench) (Harris et al., 2007). Leaf chlorophyll fluorescence (*F*_v_/*F*_m_) has been used to study changes in photosynthetic activity in response to abiotic stresses under drought or heat treatment (Li et al., 2014; Bhusal et al., 2018; Maulana et al., 2018). The ratio of variable fluorescence (*F*_v_) to maximum fluorescence (*F*_m_) is used to estimate the potential quantum efficiency of photosystem (PS) II maximum efficiency under abiotic stress conditions (Sayed, 2003; Rachmilevitch, 2006). This technique has been used as one of the stress-tolerance screening tools in different crop species including wheat (Yang et al., 2007; Kumar et al., 2012; Maulana et al., 2018), sorghum (Sayyad-Amin et al., 2016; Sukumaran et al., 2016), rice (*Oryza sativa* L.) (Lone et al., 2019), barley (*Hordeum vulgare* L.) (Li et al., 2006), and maize (*Zea mays* L.) (Song et al., 2018). Generally, a low *F*_v_/*F*_m_ value indicates low photosynthetic efficiency, whereas greater *F*_v_/*F*_m_ values under stress conditions show better tolerance to stress (Kumar et al., 2012).

The rapid development of genotyping and phenotyping technologies over the years has greatly helped the understanding of the physiological and genetic bases of polygenic traits, such as drought tolerance in wheat (Kumar et al., 2012; Edae et al., 2014; Sukumaran et al., 2018). To date, QTL mapping using bi-parental mapping population has been widely used for dissecting the genetic basis of drought tolerance in various crops, such as wheat (Kumar et al., 2012; Gahlaut et al., 2017), maize (Zhu et al., 2011), rice (Qu et al., 2008), and sorghum (Kebede et al., 2001). However, the development of mapping population is time-consuming. In addition, the bi-parental mapping approach can detect only a small portion of the genome regions contributing to the trait of interest because of the specific genetic background of the population.

In recent years, genome-wide association study (GWAS) has been used to detect QTL for abiotic and biotic stress tolerance in different crop species, including wheat (Czyczyło-Mysza et al., 2011; Gupta et al., 2017; Maulana et al., 2018). Using GWAS, Sukumaran et al. (2018) detected multiple significant QTL associated with yield and yield components of durum wheat grown under different growing conditions, including yield potential, heat, and drought stress environments. Similarly, studies detected QTL associated with seedling heat tolerance-related traits (Li et al., 2012; Maulana et al., 2018) and drought tolerance-related agronomic traits in wheat (Mwadzingeni et al., 2017; Gahlaut et al., 2019). Studies have found marker–trait associations for drought stress tolerance on different chromosomes, including 1A, 2A, 2B, 2D, 3D, 4A, 4D, 5A, and 7B, of wheat (Mwadzingeni et al., 2017; Shi et al., 2017; Sukumaran et al., 2018; Gahlaut et al., 2019; Mathew et al., 2019). However, few studies on drought tolerance of wheat have been conducted at the seedling stage. This study was aimed at mapping QTL contributing to seedling drought tolerance in winter wheat for MAS of the trait during wheat breeding.

The objectives of this study were to map QTL associated with seedling drought tolerance-related traits in winter wheat and to identify single-nucleotide polymorphism (SNP) markers that can be used for MAS of seedling drought tolerance during wheat breeding.

## Materials and Methods

### Plant Materials and Phenotyping

A total of 200 diverse representative lines, selected from the Hard Winter Wheat Association Mapping Panel (HWWAMP) of the Triticeae Coordinated Agricultural Project (TCAP) (Guttieri et al., 2015), were used in this study. Two sets of experiments, WW and DS, were conducted in a climate-controlled greenhouse at Noble Research Institute, LLC (Ardmore, OK, United States). The experiment was repeated four times. In every repeat, a total of six pots, with three biological replicates in each pot, per line were used. The pots were split into two sets (i.e., three pots for the WW and three pots for the DS) and laid in a randomized complete block design. Plants were initially planted under WW growth conditions for 21 days. At the five-leaf stage, one set of plants was subjected to DS by withdrawing water for 14 days, whereas the other set of plants continued to receive the optimum amount of water. After 14 days of DS, watering was resumed on the DS set to assess genotypic variability in seedling recovery after removal of stress.

Data were collected on leaf chlorophyll content, leaf chlorophyll fluorescence, shoot length, number of leaves per seedling, and seedling recovery after removal of DS. Ten days after DS treatment, leaf chlorophyll content and leaf chlorophyll fluorescence data were collected. A SPAD chlorophyll meter (Model 502, Spectrum Technologies, Aurora, IL, United States) and a pulse modular fluorimeter (Model OS5-FL, Opti-Sciences, Hudson, NH, United States) were used to measure leaf chlorophyll content and leaf chlorophyll fluorescence, respectively. Prior to measuring leaf chlorophyll fluorescence, leaves were dark-adapted for at least 20 min to fix a non-stressed reference point (Yang et al., 2007; Sukumaran et al., 2016). Fourteen days after DS treatment, the shoot length and number of leaves per seedling data were collected. Shoot length was recorded by measuring seedling height from the soil base to the tip of the longest leaf. The number of leaves per seedling was determined as the average number of leaves from three biological replicates in each pot. Seedling recovery was the percentage of plants that were able to recover 7 days after DS was removed, and it was calculated from the biological replicates. Drought stress response of a trait, referred to as relative difference of the trait, was calculated as the difference between the trait performance at WW and DS conditions and then divided by its performance at the WW condition.

### Genotyping and Population Structure Analysis

The association mapping panel was genotyped using the wheat 90K SNP array, generating 15,574 SNP markers after filtering with more than 5% minor allele frequency (MAF) and less than 10% missing data. Chromosomal positions of the SNPs were based on the 90K SNP consensus map (Wang et al., 2014) because many SNPs were not able to be mapped to the wheat reference sequence (IWGSC, 2018).

Population structure analysis was performed using principal component (PC) analysis in R program with the function *princomp*. The optimal number of PCs (where the “elbow” point occurred) used as a covariate in the GWAS model was determined from a scree plot generated by plotting the percentage of variances explained by the first 10 PCs against the number of PCs (Maulana et al., 2018).

### Genome-Wide Association Mapping Analysis

Genome-wide association mapping analysis was performed in the R package GAPIT (Lipka et al., 2012) using a mixed linear model (MLM) (Yu et al., 2006). This model was chosen because of its high statistical power compared with other models. With MLM, population structure (PC) and familial relatedness (K-matrix) were included as fixed- and random-effect covariates, respectively, thereby reducing false positive signals. Firstly, we declared significant QTL and SNPs based on false discovery rate (FDR < 0.05) as a cut-off point. However, the FDR was too stringent in this study, so we finally used a lower cut-off threshold using an unadjusted *p*-value of <0.001 to declare significant QTL (Mwadzingeni et al., 2017; Sukumaran et al., 2018; Mathew et al., 2019). Visualization of significant QTL and SNPs was done using Manhattan plots with the *qq*man R package (Turner, 2014).

### Candidate Gene Analysis

We performed a BLAST search to identify genes or related proteins with DNA sequences similar to those of significant SNP markers associated with seedling drought tolerance-related traits detected in the present study. The BLAST search was conducted using the wheat reference sequence hosted by the URGI-INRA (Alaux et al., 2018).

## Results

### Phenotypic Data Analysis

**FIGURE 1:**
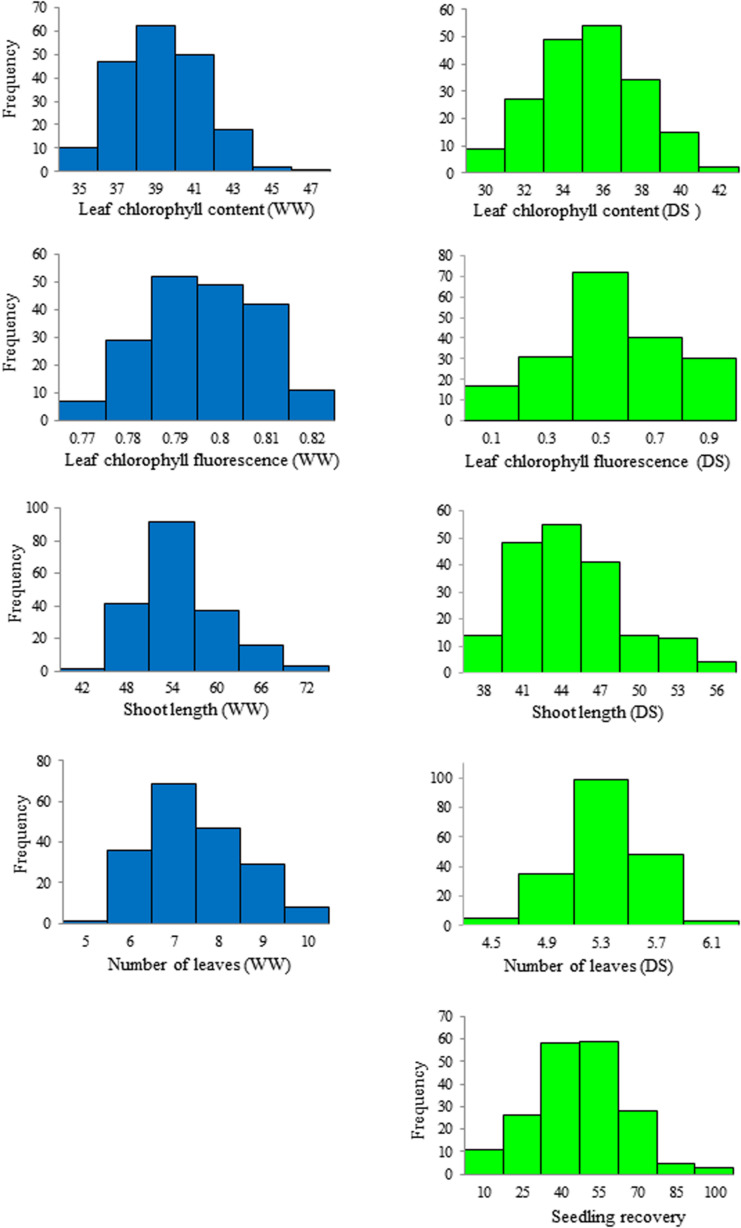
Frequency distribution of the seedling traits observed at well-watered (WW) and drought-stressed (DS) growth conditions in the wheat diversity panel.

Phenotypic variation and frequency distribution of the traits measured from the population are presented in Table 1 and Figure 1, respectively. Large phenotypic variation was observed in all traits under both WW and DS growth conditions (Table 1). For leaf chlorophyll content (SPAD), mean values were 38.3 and 34.3, ranging from 33.8 to 46.0 and from 27.1 to 42.4, under WW and DS growth conditions, respectively. For leaf chlorophyll fluorescence, there was less variation among lines under the WW growth condition, but there was large variation under the DS condition. Mean leaf chlorophyll fluorescence value was 0.79 for the WW control compared with 0.43 for plants under the DS condition. Leaf chlorophyll fluorescence ranged from 0.75 to 0.85 under the WW growth condition, compared with 0.002 to 0.77 under the DS growth condition (Table 1).

**TABLE 1:** Performance of seedling drought tolerance-related traits of the panel used in this study.

**Trait**	**Well-Watered**			**Drought stress**		
	**Mean**	**Range**	SD*	**Mean**	**Range**	SD*
Leaf chlorophyll content (SPAD)	38.3	33.8–46.0	2.19	34.3	27.1–42.4	2.61
Leaf chlorophyll fluorescence	0.79	0.75–0.85	0.01	0.43	0.002–0.77	0.21
Shoot length (cm)	52.2	41.9–70.4	5.26	43.5	36.2–56.4	4.25
Number of leaves per seedling	7	5–10	1.05	5	4–6	0.28
Seedling recovery (%)	−	−	−	39.7	0.0–87.0	17.53

*Standard deviation.

Mean shoot length was 52.2 cm, ranging from 41.9 to 70.4 cm, whereas it was 43.5 cm, ranging from 36.2 to 56.4 cm, at WW and DS growth conditions, respectively (Table 1). Mean number of leaves per seedling was 7 and 5, ranging from 5 to 10 and from 4 to 6 under WW and DS growth conditions, respectively (Table 1). Phenotypic variation of number of leaves per seedling among DS-treated lines was very small in contrast to the large variation in WW plants, because almost all plants were at the five-leaf stage when the drought treatment started, but DS-treated plants did not grow much during the 14 days drought treatment period compared with WW plants. For seedling recovery after DS, on average, 39.7% of seedlings were able to recover after the removal of DS treatment (Table 1). Generally, seedling recovery ranged from 0 to 87.0%. Overall, DS reduced leaf chlorophyll content, leaf chlorophyll fluorescence, shoot length, and number of leaves per seedling by 10.5, 45.6, 16.8, and 28.6%, respectively.

### Genome-Wide Association Mapping Analysis

**FIGURE 2:**
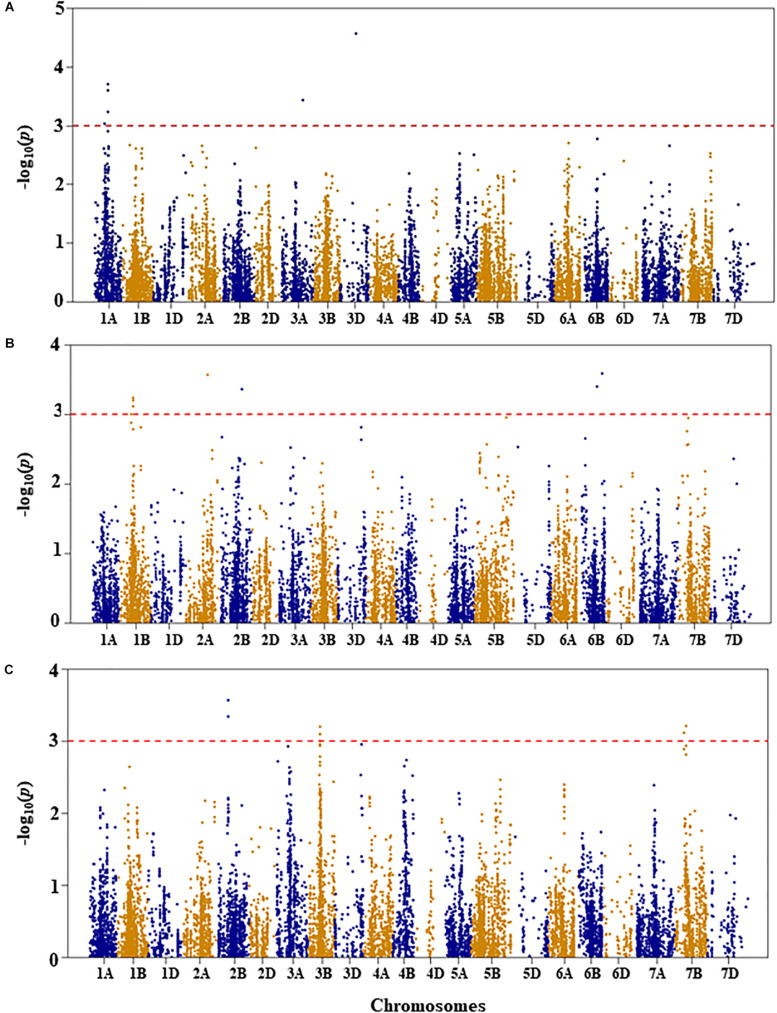
Manhattan plots of GWAS conducted on leaf chlorophyll content of the association mapping panel. **(A)** Well-watered, **(B)** drought-stressed growth condition, and **(C)** drought stress response using the trait relative difference between the two water regimes.

**FIGURE 3:**
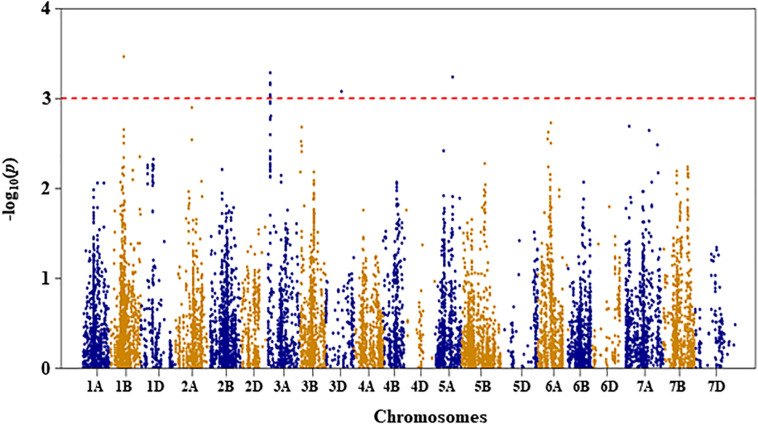
Manhattan plot of leaf chlorophyll fluorescence of the association mapping panel under drought-stressed growth condition.

**FIGURE 4:**
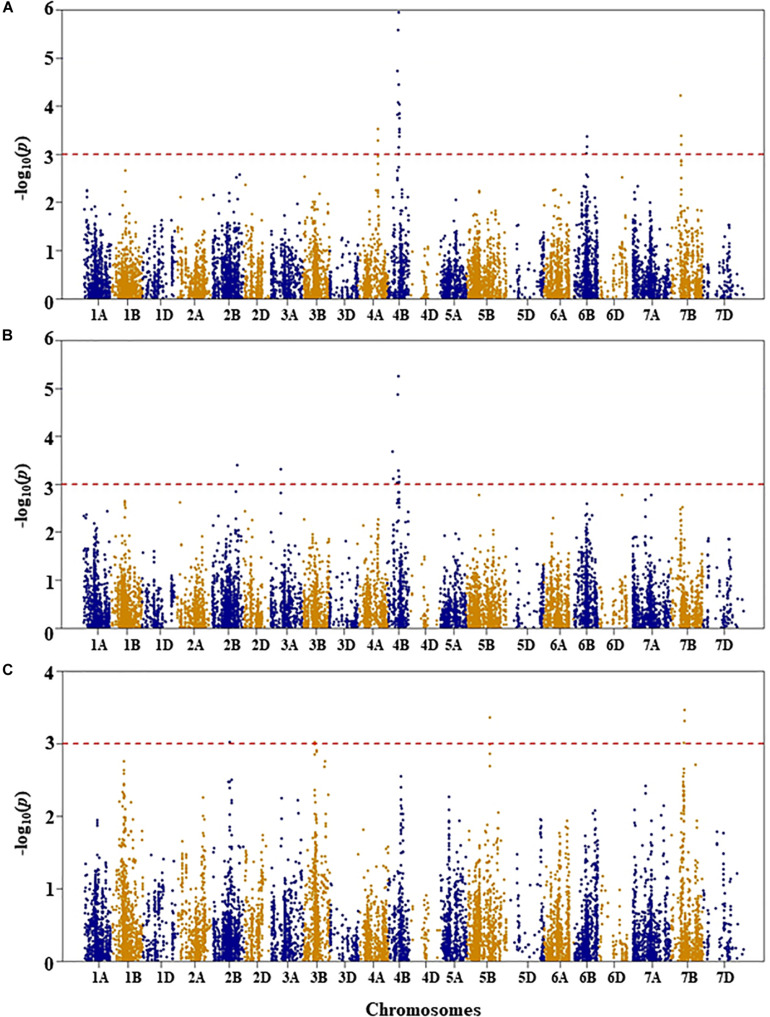
Manhattan plots of GWAS conducted on shoot length of the association mapping panel. **(A)** Well-watered, **(B)** drought-stressed growth condition, and **(C)** drought stress response using the trait relative difference between the two water regimes.

**FIGURE 5:**
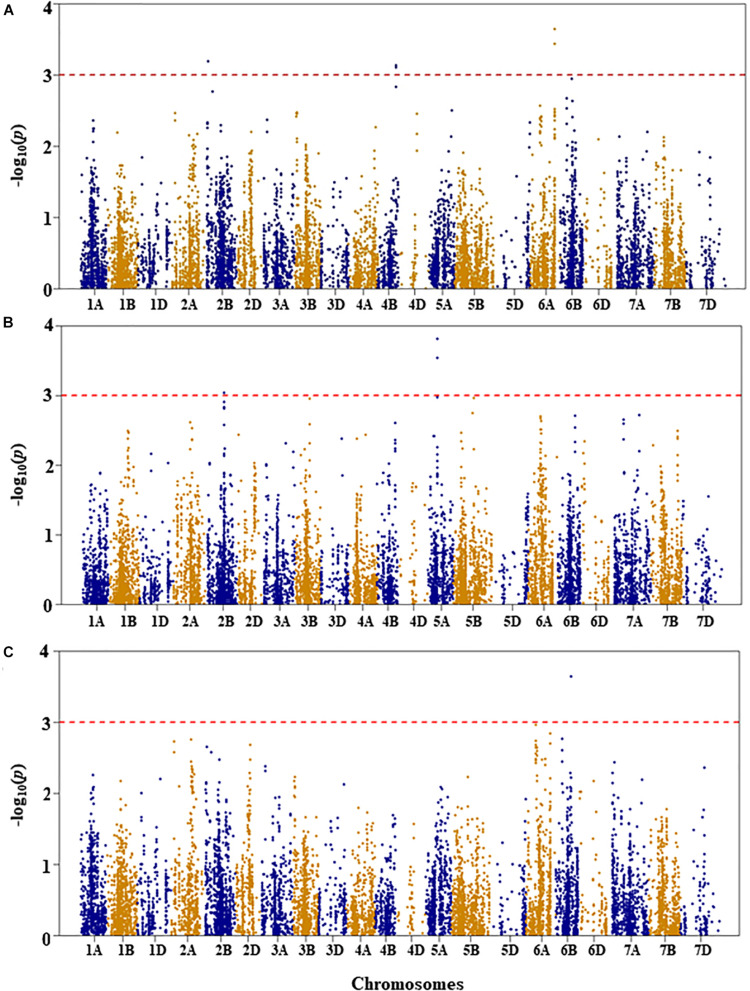
Manhattan plots of GWAS conducted on number of leaves per seedling of the association mapping panel. **(A)** Well-watered, **(B)** drought stressed growth condition, and **(C)** drought stress response using the trait relative difference between the two water regimes.

**FIGURE 6:**
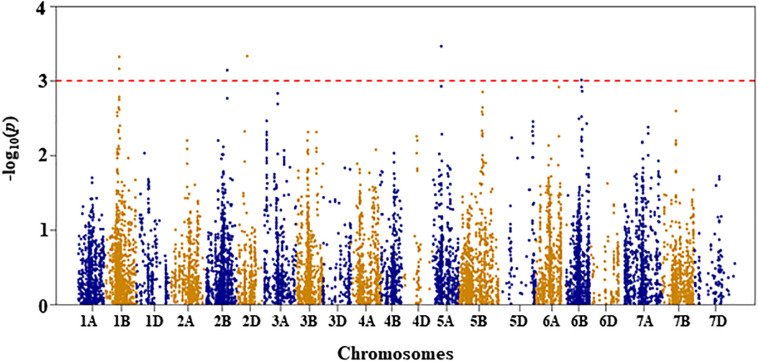
Manhattan plot of GWAS conducted on seedling recovery after removal of drought stress treatment of the association mapping panel.

Genetic diversity and population structure analyses were conducted as previously reported (Maulana et al., 2018). For GWAS analysis in the present study, we used three PCs as a fixed-effect covariate in the MLM to correct for population structure (Maulana et al., 2018). The optimal number of PCs was determined from a scree plot generated by plotting the percentage of variances explained by the first 10 PCs against the number of PCs (Maulana et al., 2018). Results of genome-wide association mapping analyses are presented in Figures 2–6. Although no QTL were declared significant at a FDR < 0.05 except for shoot length, significant SNPs were detected at an unadjusted *p*-value < 0.001 under WW and/or DS growth conditions. The QTL and SNP markers significantly associated with seedling drought tolerance-related traits under WW and DS growth conditions are presented in Table 2, with additional details given in Supplementary Table S1. In addition, for a given trait, the QTL and the SNP markers significantly associated with drought stress response of the trait, which was the relative difference of the trait between the two water conditions, are also presented.

**TABLE 2:** Drought stress responding QTL at the seedling stage of wheat in the present study.

**Trait**	**Drought Trait**	**QTL**	**Representative SNP**	**Chr**	**Position (cM)**	**R^2^ (%)**
Leaf chlorophyll content (SPAD)	Drought stress	QLCCDS.nri-1B	IWB26948	1B	76–77	6.05
		QLCCDS.nri-2A	IWB11193	2A	124	6.81
		QLCCDS.nri-2B	IWB12155	2B	129	6.32
		QLCCDS.nri-6B.1	IWB8110	6B	80	6.41
		QLCCDS.nri-6B.2	IWA5606	6B	110	6.85
	Drought stress response	QLCCDR.nri-2B	IWB53473	2B	69	7.28
		QLCCDR.nri-3B	IWB10755	3B	66	6.38
		QLCCDR.nri-7B.1	IWB26212	7B	49	6.16
		QLCCDR.nri-7B.2	IWB1369	7B	59	6.40
Leaf chlorophyll fluorescence (*F*_*v*_/*F*_*m*_)	Drought stress	QLCFDS.nri-1B	IWB38151	1B	80	7.06
		QLCFDS.nri-3A	IWB60417	3A	24–26	6.63
		QLCFDS.nri-3D	IWB58554	3D	86	6.13
		QLCFDS.nri-5A	IWA3705	5A	99	6.50
Shoot length (cm)	Drought stress	QSLDS.nri-2B	IWB58206	2B	153	4.77
		QSLDS.nri-3A	IWB49895	3A	68	4.62
		QSLDS.nri-4B.1	IWA1768	4B	30–33	5.25
	Drought stress response	QSLDR.nri-2B	IWB29525	2B	107	5.99
		QSLDR.nri-3B	IWB26799	3B	62–63	6.00
		QSLDR.nri-5B	IWB73678	5B	129	6.83
		QSLDR.nri-7B	IWB73494	7B	71–73	7.08
Number of leaves per seedling	Drought stress	QLNDS.nri-2B	IWB2982	2B	109	5.78
		QLNDS.nri-5A	IWB4229	5A	58	7.60
	Drought stress response	QLNDR.nri-6B	IWB72198	6B	83	7.40
Seedling recovery	Drought stress	QSRDS.nri-1B	IWA4680	1B	78–79	6.40
		QSRDS.nri-2B	IWB26011	2B	130	5.97
		QSRDS.nri-2D	IWA4789	2D	56	6.42
		QSRDS.nri-5A	IWB64901	5A	51	6.71
		QSRDS.nri-6B	IWB29386	6B	82	5.66

*Drought stress, QTL detected under drought stress but not at optimum water condition; drought stress response, QTL associated with drought response, which is the trait relative difference between the two water regimes. QTL were declared significant based on unadjusted *p*-value < 0.001. *R*^2^ is phenotypic variance explained by each QTL expressed as a percentage.*

#### Leaf Chlorophyll Content

For leaf chlorophyll content under WW growth condition, four QTL, represented by six SNPs, were found to be significant based on an unadjusted *p*-value < 0.001 on chromosomes 1A, 3A, and 3D (Figure 2A and Supplementary Table S1). Two QTL, *QLCCWW.nri-1A.1* and *QLCCWW.nri-1A.2*, were mapped at 65 and 84 cM, respectively, on chromosome 1A and together accounted for 11.2% of the total phenotypic variation in leaf chlorophyll content under WW growth condition. The third and fourth QTL regions (*QLCCWW.nri-3A* and *QLCCWW.nri-3D*) were mapped at the genetic distance positions of 129 and 86 cM on chromosomes 3A and 3D, respectively, and together they explained 13.7% variation of leaf chlorophyll content under WW growth condition. The most significant SNP marker (IWB58554, 86 cM) on chromosome 3D accounted for 8.0% of the total phenotypic variation of leaf chlorophyll content under WW growth condition.

For leaf chlorophyll content under DS growth condition, five QTL were found on chromosomes 1B, 2A, 2B, and 6B (Figure 2B and Supplementary Table S1). On chromosome 1B, one QTL region (*QLCCDS.nri-1B*) contributing 6.1% of the total phenotypic variation was detected by six SNPs at 76–77 cM. On chromosome 2A, one QTL, *QLCCDS.nri-2A* (124 cM), showed 6.8% variation of the trait. Similarly, on chromosome 2B, a single QTL, *QLCCDS.nri-2B* (129 cM), was found explaining 6.3% variation in leaf chlorophyll content under DS growth condition. On chromosome 6B, two QTL (*QLCCDS.nri-6B.1* and *QLCCDS.nri-6B.2*) were mapped at 80 and 110 cM, respectively. The two QTL together explained about 13.3% of the total phenotypic variation in leaf chlorophyll content. The most significant SNP markers associated with leaf chlorophyll content under DS growth condition were IWA5606 and IWB36649, co-localized at 110 cM on chromosome 6B (Supplementary Table S1).

For DS response of the leaf chlorophyll content, i.e., the relative difference in leaf chlorophyll content under the two water regimes, four significant QTL were identified on chromosomes 2B, 3B, and 7B (Figure 2C and Supplementary Table S1). One QTL (*QLCCDR.nri-2B*) was identified by two significant SNP markers at 69 cM on chromosome 2B, and it accounted for about 7.3% of the total phenotypic variation of DS response for leaf chlorophyll content (Supplementary Table S1). A QTL (*QLCCDR.nri-3B*) causing 6.4% of leaf chlorophyll content variation in response to DS was also mapped at a genetic position of 66 cM on chromosome 3B. On chromosome 7B, two QTL, *QLCCDR.nri-7B.1* (49 cM) and *QLCCDR.nri-7B.2* (59 cM), were identified, and together they explained 12.6% of the total phenotypic variation in DS response for leaf chlorophyll content. Overall, the most significant SNP markers associated with DS response for leaf chlorophyll content were IWB53473 and IWB31701 on chromosome 2B.

In short, the data suggest that the leaf chlorophyll content QTL associated with drought stress or drought response are located on chromosomes 1B, 2A, 2B, 3B, 6B, and 7B based on the QTL detected for DS response of the trait or the QTL detected under DS but not under WW condition (Table 2). In fact, there was no common leaf chlorophyll content QTL detected for the two water treatments.

#### Leaf Chlorophyll Fluorescence

For leaf chlorophyll fluorescence, marker–trait association was only conducted for the trait measured under DS condition because the variation of the trait was too small under WW condition (as shown in Table 1). Four QTL, represented by 11 SNP markers on chromosomes 1B, 3A, 3D, and 5A, were significantly associated with the trait under DS (Figure 3 and Supplementary Table S1). The first QTL (*QLCFDS.nri-1B*) on chromosome 1B (80 cM) explained 7.1% of the total phenotypic variation in leaf chlorophyll fluorescence under DS growth condition. The second QTL (*QLCFDS.nri-3A*) was on chromosome 3A within the genomic region spanning from 24 to 26 cM. This QTL was detected by eight SNPs that caused about 6.6% variation in leaf chlorophyll fluorescence under DS growth condition. The third QTL (*QLCFDS.nri-3D*) region was located at 86 cM on chromosome 3D, explaining about 6.1% variation of the trait. The fourth QTL (*QLCFDS.nri-5A*) was located on chromosome 5A at the genetic distance of 99 cM, which accounted for 6.5% of the total phenotypic variation of the trait. The most significant SNP markers were IWB38151 (80 cM) and IWB60417 (24 cM) on chromosomes 1B and 3A, respectively (Table 2).

#### Shoot Length

For shoot length under WW condition, five QTL were identified by 25 SNPs at an unadjusted *p*-value < 0.001 significance on chromosomes 4A, 4B, 6B, and 7B (Figure 4A and Supplementary Table S1). The first QTL (*QSLWW.nri-4A*) was mapped at 111 cM on chromosome 4A, explaining 4.7% of the total phenotypic variation in shoot length under WW growth condition. On chromosome 4B, two QTL regions (*QSLWW.nri-4B.1* and *QSLWW.nri-4B.2*) were mapped at 54–58 and 60–64 cM, for 8.2 and 8.8%, respectively, of the total phenotypic variation in shoot length. The other QTL (*QSLWW.nri-6B*) at 64–67 cM on 6B accounted for 4.5% variation in shoot length under WW growth condition. On chromosome 7B, *QSLWW.nri-7B* (54–58 cM) caused 5.8% variation of the trait. Overall, the most significant SNPs associated with shoot length under WW condition were IWB12856 (60 cM) and IWB35611 (57 cM) on chromosome 4B (Supplementary Table S1).

For shoot length under DS growth condition, five QTL represented by 11 SNPs were found on chromosomes 2B, 3A, and 4B (Figure 4B and Supplementary Table S1). On chromosome 2B, the QTL (*QSLDS.nri-2B*) was mapped at 153 cM, explaining 4.8% of the total phenotypic variation in shoot length. The QTL (*QSLDS.nri-3A*) on chromosome 3A (68 cM) accounted for 4.6% variation in shoot length. In addition, three QTL (*QSLDS.nri-4B.1*, *QSLDS.nri-4B.2*, and *QSLDS.nri-4B.3*) were detected on chromosome 4B. The *QSLDS.nri-4B.1* was mapped at 30–33 cM, and it explained 5.3% of the total phenotypic variation of the trait. The *QSLDS.nri-4B.2* at 54–57 cM accounted for 7.4% variation of the trait. The *QSLDS.nri-4B.3* at 60–64 cM caused 8.1% of the total phenotypic variation of the trait. Overall, the most significant SNPs associated with shoot length under DS growth condition were IWB12856 (60 cM) and IWB35611 (57 cM) on chromosome 4B (Supplementary Table S1).

For DS response of shoot length, four QTL were detected on chromosomes 2B, 3B, 5B, and 7B (Figure 4C and Supplementary Table S1). One QTL (*QSLDR.nri-2B*) was mapped at 107 cM on 2B, explaining 6.0% of the total phenotypic variation in DS response of shoot length. The second QTL (*QSLDR.nri-3B*) at 62–63 cM on 3B also explained 6.0% variation of the trait. The third QTL region (*QSLDR.nri-5B*) at 129 cM on 5B was responsible for 6.8% of the total phenotypic variation in DS response for the trait. The fourth QTL region (*QSLDR.nri-7B*) at 71–73 cM on 7B caused 7.1% variation of the trait.

Overall, the data suggest that drought stress or drought response QTL contributing to shoot length are located on chromosomes 2B, 3A, 3B, 4B, 5B, and 7B based on QTL detected for drought stress response of the trait or QTL detected under DS but not WW growth condition (Table 2). The QTL at 60–64 cM on 4B detected commonly under both WW (*QSLWW.nri-4B.2*) and DS (*QSLDS.nri-4B.3*) conditions should not contribute to drought stress.

#### Number of Leaves per Seedling

For number of leaves per seedling under WW growth condition, three QTL were detected on chromosomes 2B, 4B, and 6A at genetic positions of 21, 105, and 141 cM, respectively (Figure 5A and Supplementary Table S1). The three QTL (*QLNWW.nri-2B*, *QLNWW.nri-4B*, and *QLNWW.nri-6A*) explained about 6.4, 6.2, and 7.4, respectively, of the total phenotypic variation in number of leaves per seedling under WW growth condition.

Under DS growth condition, two QTL significantly associated with number of leaves per seedling were detected on chromosomes 2B and 5A at genetic positions of 109 and 58 cM, respectively (Figure 5B and Supplementary Table S1). The *QLNDS.nri-2B* and *QLNDS.nri-5A* were responsible for 5.8 and 7.6%, respectively, of the total phenotypic variation in number of leaves per seedling under DS growth condition. Overall, the most significant SNP was IWB4229, followed by IWB14493 on chromosome 5A.

For DS response of number of leaves per seedling, a QTL (*QLNDR.nri-6B*) was found on chromosome 6B (Figure 5C and Supplementary Table S1). This QTL was represented by two SNPs co-localized at the genetic distance of 83 cM, and it accounted for 7.4% of the total phenotypic variation in DS response of number of leaves per seedling.

The overall data indicate that drought responding QTL associated with number of leaves per seedling are located on chromosomes 2B, 5A, and 6B according to QTL detected for drought stress response of the trait or QTL detected under DS in contrast to WW growth condition (Table 2).

#### Seedling Recovery

For seedling recovery after removal of DS treatment, five QTL, represented by six SNPs, were detected on chromosomes 1B, 2B, 2D, 5A, and 6B (Figure 6 and Supplementary Table S1). These five QTL, *QSRDS.nri-1B*, *QSRDS.nri-2B*, *QSRDS.nri-2D*, *QSRDS.nri-5A*, and *QSRDS.nri-6B*, located at 78–79, 130, 56, 51, and 82 cM on corresponding chromosomes explained 6.4, 6.0, 6.4, 6.7, and 5.7%, respectively, of the total phenotypic variation in seedling recovery after DS (Table 2).

## Discussion

Planting time is one of the most critical factors to consider when growing dual-purpose winter wheat for increased autumn–winter forage and GY. In the southern Great Plains of the United States, wheat intended for winter grazing needs to be planted in early September for increased autumn–winter forage production (Kumssa et al., 2019). However, the crop often faces an establishment challenge because of inadequate amounts of soil moisture and high air temperatures during the seedling stage. Poor seedling establishment can reduce not only autumn–winter forage production for cattle grazing but also GY by the end of the season (Maulana et al., 2019). Therefore, breeding for improved tolerance to seedling drought stress is critical for sustainable production of forage and grain in dual-purpose winter wheat. However, phenotyping drought tolerance at the seedling stage is difficult under field conditions. MAS provides a complementary approach to address this limitation. A GWAS was conducted to map QTL and identify SNP markers associated with seedling drought tolerance for marker-assisted breeding of the trait. Discovery of QTL or genetic markers associated with seedling drought tolerance will facilitate the development of seedling drought-tolerant wheat cultivars through MAS.

In the association mapping panel evaluated in this study, we observed significant phenotypic variation among lines in seedling drought tolerance-related traits, including leaf chlorophyll content, leaf chlorophyll fluorescence, shoot length, number of leaves per seedling, and seedling recovery under both WW and DS growth conditions. This result suggests that there are genetic sources of seedling drought tolerance in the panel used in this study. Our results agree with a previous study done using the same association panel from TCAP (Awad, 2015). The author evaluated the TCAP panel in multiple environments differing in soil moisture for agronomic and drought tolerance-related traits and found significant phenotypic variation among wheat lines in the panel with regard to drought tolerance at the reproductive and/or grain-filling stages.

In the past, most QTL studies for drought tolerance in wheat have been conducted at the flowering and/or grain-filling stages (Kirigwi et al., 2007; Li et al., 2015; Gahlaut et al., 2017; Shi et al., 2017; Sukumaran et al., 2018), however, few reports about QTL for seedling drought tolerance are available (Lin et al., 2019). In this study, we used MLM to map QTL and identify SNP markers associated with seedling drought tolerance-related traits. We identified multiple significant QTL, spread across the genome, associated with seedling drought tolerance-related traits under both WW and DS growth conditions. To identify QTL associated with seedling drought tolerance, we compared QTL identified under DS vs. WW growth conditions because QTL identified commonly under both DS and WW conditions are not relevant to drought stress. In addition, drought stress response QTL were mapped using the relative performance of each trait between the WW and DS growth conditions.

Physiological traits, leaf chlorophyll content and chlorophyll fluorescence, have been used as good indicators of the photosynthetic capacity of the plant in different crops (Harris et al., 2007; Kumar et al., 2012; Lopes and Reynolds, 2012). In the present study, QTL associated with leaf chlorophyll content under DS were located on chromosomes 1B, 2A, 2B, 3B, 6B, and 7B. Our results corroborate other QTL studies done in wheat at the adult plant stage, where QTL associated with leaf chlorophyll content under drought stress were detected on chromosomes 1A, 1B, 2B, 3A, 3B, 4A, 4B, 5A, 5B, 6A, 6B, 7A, and 7D (Yang et al., 2007; Peleg et al., 2009; Kumar et al., 2012; Barakat et al., 2015; Shi et al., 2017). However, in the present study, we did not find QTL for leaf chlorophyll content under DS condition or DS response on chromosomes 1A, 3A, 4A, 4B, 5A, 5B, 6A, 7A, and 7D, but we detected a novel QTL on chromosome 2A that was not reported in the previous QTL studies mentioned above.

In addition, QTL for leaf chlorophyll fluorescence under DS growth condition were identified on chromosomes 1B, 3A, 3D, and 5A. However, previous QTL studies (Yang et al., 2007; Kumar et al., 2012) under DS in wheat found QTL for leaf chlorophyll fluorescence on several other chromosomes that were not detected in our study. The QTL *QLCFDS.nri-3D* for leaf chlorophyll fluorescence on chromosome 3D co-localized with QTL for GY of the same population from a separate study under rain-fed field condition (as drought stress treatment) (Awad, 2015).

Furthermore, BLAST search showed that some of the significant SNP markers identified in the present study aligned with candidate genes, known to be involved in plant stress responses, such as salinity, drought, heat, and cold tolerance in different crops, including wheat. For example, on chromosome 1B, multiple significant SNPs found to be associated with leaf chlorophyll content under DS condition in the present study have 93–99% DNA sequence similarities with DNA replication licensing factor MCM3, which was reported to play an important role in salt and cold stress tolerance in other crop species, such as pea (*Pisum sativum* L.) and brassica (*Brassica rapa* L.) (Shultz et al., 2009; Tuteja et al., 2011; Supplementary Table S1). On chromosome 3A, significant SNP IWB60417 for leaf chlorophyll fluorescence under the DS growth condition has 96% sequence similarity with the candidate gene, DExH-box ATP-dependent RNA helicase DExH3-like. This gene was reported to be involved in plant development and abiotic stress tolerance, such as plant chilling and freezing tolerance (Liu and Imai, 2018). Moreover, the significant SNP, IWB11846, for leaf chlorophyll fluorescence under the DS condition on chromosome 3A has 99% sequence similarity with the fructose-1,6-bisphosphate aldolase 1 (FBA1) gene. This gene plays a role in diverse plant stress responses, such as drought and temperature stress tolerance in *Arabidopsis* (Lu et al., 2012).

For morphological traits, QTL associated with shoot length under DS but not under WW were detected in chromosomes 2B and 3A, whereas QTL associated with drought stress response for shoot length were found in chromosomes 2B, 3B, 5B, and 7B (Table 2). Our results corroborate previous QTL studies for DS tolerance-related traits at the flowering or grain-filling stage of wheat on chromosomes 1A, 1B, 2A, 2B, 2D, 3A, 4B, 5B, 5D, 6A, 6B, 7A, and 7B (Mwadzingeni et al., 2017; Sukumaran et al., 2018). However, some QTL detected in previous studies were not found in the present study. Similarly, for number of leaves per seedling and seedling recovery, some of the QTL detected in this study were located on the same chromosomes that were reported in other DS studies at various adult plant stages (Li et al., 2015; Gahlaut et al., 2017; Shi et al., 2017). Furthermore, under DS growth conditions, *QLNDS.nri-5A* for number of leaves co-localized with QTL for canopy temperature at the grain-filling stage of the same panel evaluated under rain-fed field conditions. This QTL was represented by two significant SNP markers, IWB14493 (CAP_c1066_309) and IWB4229 (BobWhite_c6759_365), and mapped at the genetic distance position of 58 cM on chromosome 5A (Awad, 2015).

Recently, wheat morphological traits at the seedling stage were also mapped in a different population under drought stress (Lin et al., 2019). The study detected QTL associated with various seedling root and shoot traits on different chromosomes, some of which corroborates the chromosomes 2B, 5A, and 6B that were detected in the present study.

In addition to locating major QTL based on means, in this study, GWAS was also conducted with individual repeats as a reference for locating QTL that were relatively stable across different experimental repeats that might involve variation in growth conditions. For this purpose, we highlighted those QTL detected using means as stable QTL if the same QTL were also detected in at least two experimental repeats (Supplementary Table S1). In total, we detected 12 stable QTL responding to drought stress, including two for leaf chlorophyll content (*QLCCDR.nri-3B* and *QLCCDS.nri-6B.1*), four for leaf chlorophyll fluorescence (*QLCFDS.nri-1B*, *QLCFDS.nri-3A*, *QLCFDS.nri-3D*, and *QLCFDS.nri-5A*), four for shoot length (*QSLDS.nri-2B*, *QSLDS.nri-3A*, *QSLDS.nri-4B.1*, and *QSLDS.nri-4B.2*), and two for number of leaves per seedling (*QLNDS.nri-5A* and *QLNDR.nri-6B*). The results indicated that shoot length and leaf chlorophyll fluorescence were good indicators in responding to drought stress because most of the drought responding QTL detected using means of these two traits were also detected in at least two individual repeats (Supplementary Table S1). Notably, under WW growth condition, most QTL detected for shoot length using means were also repeatable in individual experimental repeats (Supplementary Table S1), again indicating that shoot length was the most reliable seedling trait. The QTL associated with shoot length were more stable across growth conditions. These stable QTL are much more valuable for use in MAS during wheat breeding.

Different physiological and morphological traits were mapped to common chromosomes, and QTL regions associated with two or more studied traits were located on 1B, 2B, 3B, and 6B in the present study (Supplementary Table S2). For example, a cluster of multiple QTL associated with leaf chlorophyll content, number of leaves per seedling, shoot length, and seedling recovery was mapped at the genetic position of 107–130 cM on chromosome 2B. A previous study also found a cluster of QTL for drought stress indices of grain-related traits on chromosome 2B (Sukumaran et al., 2018). Similarly, a cluster of QTL for leaf chlorophyll content, seedling recovery, and number of leaves was mapped at 80–83 cM on chromosome 6B (Supplementary Table S2).

Overall, the largest number of QTL for seedling drought tolerance-related traits was detected on the B genome, followed by the A genome, whereas the D genome had the lowest number of QTL. The lower number of QTL on the D genome is attributed to the lower polymorphic marker coverage compared with the A and B genomes. Similar observation has been reported in previous QTL studies done in wheat (Allen et al., 2013; Ling et al., 2013; Wu et al., 2015). Significant QTL and SNP markers identified in this study will be further validated and used in MAS of seedling drought tolerance to facilitate selection of the trait during dual-purpose wheat breeding.

## Data Availability Statement

The datasets presented in this study can be found in online repositories. The names of the repository/repositories and accession number(s) can be found in the article/ Supplementary Material.

## Author Contributions

FM phenotyped the association mapping panel, analyzed both phenotypic and genotypic data, and drafted the manuscript. WH helped in phenotyping and reviewing the manuscript. JDA assisted in experiment implementation and review of the manuscript. X-FM supervised the study and finalized the manuscript. This manuscript has been read and approved by all the authors.

## Conflict of Interest

The authors declare that the research was conducted in the absence of any commercial or financial relationships that could be construed as a potential conflict of interest.
